# Modeling Innate Immune Response to Early *Mycobacterium* Infection

**DOI:** 10.1155/2012/790482

**Published:** 2012-12-09

**Authors:** Rafael V. Carvalho, Jetty Kleijn, Annemarie H. Meijer, Fons J. Verbeek

**Affiliations:** ^1^Leiden Institute of Advanced Computer Science, Leiden University, Niels Bohrweg 1, 2333 CA Leiden, The Netherlands; ^2^Institute of Biology, Leiden University, Einsteinweg 55, 2333 CC Leiden, The Netherlands

## Abstract

In the study of complex patterns in biology, mathematical and computational models are emerging as important tools. In addition to experimental approaches, these modeling tools have recently been applied to address open questions regarding host-pathogen interaction dynamics, including the immune response to mycobacterial infection and tuberculous granuloma formation. We present an approach in which a computational model represents the interaction of the *Mycobacterium* infection with the innate immune system in zebrafish at a high level of abstraction. We use the Petri Net formalism to model the interaction between the key host elements involved in granuloma formation and infection dissemination. We define a qualitative model for the understanding and description of causal relations in this dynamic process. Complex processes involving cell-cell or cell-bacteria communication can be modeled at smaller scales and incorporated hierarchically into this main model; these are to be included in later elaborations. With the infection mechanism being defined on a higher level, lower-level processes influencing the host-pathogen interaction can be identified, modeled, and tested both quantitatively and qualitatively. This systems biology framework incorporates modeling to generate and test hypotheses, to perform virtual experiments, and to make experimentally verifiable predictions. Thereby it supports the unraveling of the mechanisms of tuberculosis infection.

## 1. Introduction

Tuberculosis (TB) is an infectious disease responsible for 1.5 million deaths annually. About one-third of the world's population is infected with the pathogen that causes this disease, *Mycobacterium tuberculosis* (*Mtb*). Most infections are controlled by the host's immune system and remain asymptomatic. However, the *Mtb* is capable to persist in the host inside granulomas, highly organized structures characterized by the presence of differentiated macrophages, lymphocytes, and other immune cells that contain, but fail to eradicate, the pathogen [1, 2]. The key to success of *Mtb* infection lies, at least in part, with the ability of the bacteria to proliferate inside host macrophages despite the antimicrobial properties of these cells. Some of the infecting bacteria can survive for extended periods within macrophages and in a granuloma, establishing long-term infections that may resurface later, for example, when the host's immune system is compromised due to malnutrition, HIV coinfection, or immunosuppressive treatment. Insight in the mechanisms that contribute to this long and complex relationship between the pathogen and the host is essential to the understanding of the fundamental aspects of TB [3].

Various animal models are used to mimic *Mtb* pathogenesis in humans, each having their specific strengths as well as limitations. In the recent years, the zebrafish has emerged as a valuable addition to the mammalian models. They are genetically tractable and have an immune system with innate and adaptive branches, very similar to the human immune system. A particularly useful property is the transparency of the embryos, which allows for real-time imaging of the interaction between pathogens and host immune cells [4–7]. *Mycobacterium marinum (Mm)*, one of the closest relatives of *Mtb*, is used to study mycobacterial pathogenesis in zebrafish. It causes a systemic tuberculosis-like infection in zebrafish, with the formation of structured granulomas that closely resemble those in human TB. The use of this model has recently contributed important insights into the function of the granuloma in expansion and dissemination of mycobacteria during the early stages of infection [8].

Mathematical and computational modeling provides an important additional avenue for the further exploration of disease dynamics and offers powerful and complementary tools for the study of the host-pathogen interaction. Gathering and analyzing the information from the animal model in a computational modeling process makes it possible to describe, simulate, analyze and predict the mechanism and interactions behind the infection process in intuitive and easily analyzable terms. The agent-based model (ABM) is a computational formalism based on rules that govern autonomous agents [9]; it can be used to model discrete as well as stochastic events in biology. Pappalardo et al. have implemented and simulated models using ABM and cellular automata to study the vaccine administration and immune response to cancer in mice [10–12]. Kirschner et al. have utilized ABM to model and simulate the *Mtb* disease and the host-pathogen interaction [13–15]. They suggest the ABM as an appropriate method for exploring complex spatiotemporal systems such as granuloma formation [16]. The Petri net (PN) formalism is another method providing a natural and promising modeling technique useful for modeling metabolic pathways and biological behavior [17]. The PN formalism is, typically, very suitable for systems with a concurrent nature, that is, systems in which processes occur in parallel. In essence, the PN is a mathematical modeling language based on a directed bipartite graph. The PN formalism has already been successfully applied on case studies in biology to create, verify, and validate models. The stochastic activity network (SAN) is an extended Petri net model that uses probabilistic time and is in particular useful for performance evaluation. Tsavachidou and Liebman [18] have used SAN in modeling and quantitative evaluation of the biological pathways involved in menopause. They use biological pathways and experimental data in an accurate quantitative model to simulate and compare to *in vivo/in vitro* experiments. Peleg et al. [19] have used colored hierarchical PNs to study the effects of mutations in tRNA on the protein translation. They define qualitative models of molecular function at different levels of granularity. The application domain of tRNA was chosen due the abundant literature on tRNA molecular structure as well as the diseases that relate to abnormal structure. Regarding the process of mycobacterial infection, the interaction with host-pathogen is complex and much remains unknown and significance of specific immune factors present on the mycobacterial infection process still poorly understood. To date, mathematical and computational models applied to mycobacterial infection have been used to explore specific aspects at various biological scales (e.g., intracellular, cell-cell interactions, and cell population dynamics) [14–16]. The mycobacterial infection process thus is composed of numerous subprocesses, some of which are mutually dependant, giving rise to a very complex set of interactions. A model describing the process at a higher level is missing, and therefore we take the construction of a model of the infection mechanism at a higher level of granularity as a starting point for our modeling efforts and explorations. The availability of such a model enables to connect and visualize the whole infection process. This top-down approach allows identifying, modeling, and testing of the lower-level processes in both qualitative and quantitative manner. The input for these lower-level processes is obtained from both empirical research and literature *data.*

The* zebrafish* model of *Mycobacterium* infection, based on *Mm* infection, has been identified as very useful in the understanding of host-mycobacteria interactions during early stages of infection. This model system is used to generate experimental data that elucidate the pathogenesis as well as to transfer the findings to the human case. The perspective of analysis from* in vivo/in vitro *studies requires an integration layer so that experimental data can be understood in the range of complex interactions that are underlying the infection process. Therefore, we intend to construct such integration layer from an *in silico* perspective using the Petri net formalism as a modeling method to simulate bacteria-host interactions in early stages of tuberculous granuloma formation. As indicated, our starting point is to construct such a model from a higher level of abstraction. We, therefore, designed a PN by first identifying the processes in the infection process, that is, phagocytosis of mycobacteria by macrophages, the migration of infected macrophages to deeper tissue, the growth of mycobacteria within individual macrophages, and the granuloma formation and maturation. These processes were represented in a qualitative colored Petri net (CPN) using the Snoopy software, a tool for modeling and animating/simulating hierarchical graph-based formalisms. The information analysis on the processes was obtained from recent literature about the phases involved in the early response to mycobacterial infection [8] and from interviews with researchers.

From the processes as the major design elements, we constructed a qualitative colored Petri net on a level of abstraction that helps understanding and describing the causal relations in a dynamic process. In addition to the processes, we acknowledged entities such as the zebrafish, the macrophage, the granuloma, and the bacteria. As such, the phases of the infection process are addressed whilst, for the moment, time and probability are not considered. In this manner, our model explores the disease on a high level of abstraction, modeling the factors that are crucial to visualize the mycobacterial infection process and the early immune response. Complex processes involving cell-cell or cell-bacteria communication can be modeled in a small-scale process and incorporated into the model as a hierarchical layer. As intended, the model shows the cause-effect relations that trigger the infection process. The graphical representation of the CPN communicates that in a manner a biologist can grasp immediately. Now, as the model incorporates the process of infection, the toolbox of the biologist is extended with an approach that allows to perform “what-if” as part of the experimentation whereas at the same time new experimental findings can be added to the model in a close collaboration between empirical and modeling scientists.

Starting from the abstract model of the global infection process, future extensions, such as submodels representing processes on tissue, cellular, and molecular scale, will hierarchically connect as a single model. In close collaboration with the empirical scientist and using the model, we intend to perform *in silico* experiments that are otherwise impractical or not feasible *in vivo* or *in vitro*, thereby predicting results of new experiments and generate further hypotheses about the immune system response to mycobacterial infection. The CPN model presented in this paper is the cornerstone of that process.

The remainder of this paper is structured as follows. In Section 2, we discuss the pathogenesis of the *Mycobacterium* infection in Zebrafish in more detail and next we introduce the building blocks of the CPN and the software that we have used to build the model. In Section 3, we provide a series of design considerations to come to an implementation of the model. Finally in Section 4, we end with the conclusion and discussion.

## 2. Materials and Methods

### 2.1. The Zebrafish Model of Mycobacterial Pathogenesis

**Figure 1:**
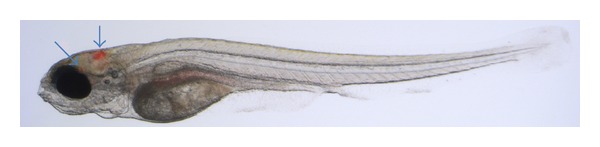
Microscope image of a zebrafish larva infected with *Mycobacterium marinum* by injection used for the study on infection progression and immune system response. Image is obtained with a Leica stereo fluorescence microscope commonly used in zebrafish research. Here the microscope image is depicted with an overlay of a fluorescent channel (red) in which the bacteria are visualized. The arrows indicate granulomas that have been developed after an induced infection with *Mycobacterium marinum. *

The zebrafish is naturally susceptible to infections caused by *M. marinum *(*Mm*), genetically closely related to *M. tuberculosis *(*Mtb*). The *Mm* infection shares pathological hallmarks with *Mtb* infection. Like other pathogenic mycobacteria, *Mm *causes chronic infection of macrophages resulting in tuberculous granulomas, making it a useful model to study mycobacterial pathogenesis [20]. Zebrafish embryos have functional innate immune cells (macrophages and neutrophils), while their adaptive immune system is not yet functional. The experimental infection of zebrafish embryos is initiated by injected bacteria into the blood circulation or into tissue. Macrophages that are attracted to the site of infection take up the mycobacteria by a process called phagocytosis. Real-time imaging of infected zebrafish embryos has allowed the direct observation of the arrival of phagocytes at the infection site and their uptake of bacteria. The macrophages are the primary cell type infected with *Mm*; however, also infected neutrophils have been observed [6, 8] and were recently shown to play an important role in *Mm* infection control [21]. In Figure 1, an *Mm* infection in a zebrafish is depicted.

Inside the macrophage, bacteria can be exposed to bactericidal mechanisms and degraded in lysosomes. However, intracellular mycobacteria are predominantly distributed between the early and late phagosomal compartments, with some also escaping into the cytoplasm [22, 23]. Similar to *Mtb*, *Mm* escapes from lysosomal degradation and its survival inside macrophages is facilitated through the dynamic modulation of a range of cellular processes. These include inhibition of pathways involved in the fusion of the phagosome with lysosomes, antigen presentation, apoptosis, and the activation of bactericidal responses [23–25]. Mycobacterial interference with the host signaling machinery severely compromises the immune defences, and the multiplication of mycobacteria inside the macrophage over time causes its death, thereby enabling further spreading of the infection. 

Once it has become infected with mycobacteria, the macrophage starts to induce recruitment of uninfected macrophages. Studies have established an important role for a mycobacterial virulence factor, the ESX-1 secretion system, in the recruitment of new macrophages to granulomas and the expansion of infected macrophages [5, 25, 26]. These macrophages efficiently find and phagocytose infected macrophages and bacteria that are released from dead cells, but in this process these macrophages are getting infected too. The aggregated macrophages become activated, a transformation reflected by an increase in their size and subcellular organelles, ruffled cell membranes, and enhanced phagocytic and microbicidal capabilities. A common feature of all *Mycobacterium* granulomas is the further differentiation of the macrophages into epithelioid cells that have tightly interdigitated cell membranes in zipper-like arrays linking adjacent cells. Those aggregates grow into organized structures that are referred to as granulomas, lumps of immune cells that surround the infection [23].

**Figure 2:**
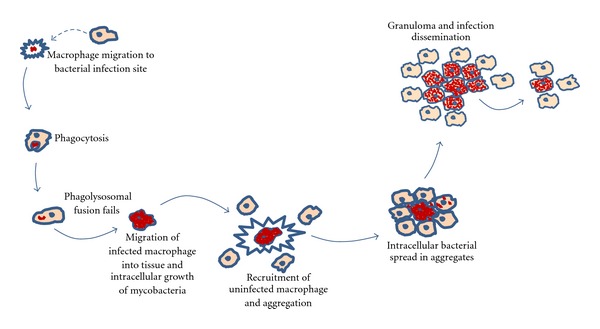
Schematic representation of the early stages of the immune response to the early stages of the mycobacterial infection process. This figure is an authors' rendition adapted from [27].

Primary granulomas are capable of disseminating infection throughout the body by egression of infected macrophages which suggests that granuloma macrophages constitute the major mechanism for dissemination of the infection [5]. These granulomas are the hallmark of the tuberculosis disease in both human and animal models. In Figure 2, a schematic representation is depicted of the early stages of the mycobacterial of the pathogenesis infection process. 

### 2.2. Computational Modeling

Experimental research has generated a tremendous amount of insights into host-pathogen interactions that occur during mycobacterial infections. Mathematical and computational models can offer powerful and complementary methods in support for better understanding the mechanisms behind the infection process in intuitive and easily analyzable terms. Amongst these methods, we can refer to modeling approaches such as Brane calculi [28], *π*-calculus [29], agent-based modeling (ABM) [16], and petri nets (PNs) [30]. These modeling methods can be used to describe, simulate, analyze, and predict the behavior of biological system by turning what is known about the biology into equations and/or rules to describe and ultimately understand the system. Previously, we proposed a system for modeling, simulating, and visualizing the *Mycobacterium *infection and granuloma formation, addressing the basic layout and the modeling challenges for this approach and evaluating between computational methods the Petri net as an appropriate method for the modeling of the infection process [31].

The Petri nets provide a formal and clear representation of systems based on their firm mathematical foundation for the analysis of system properties. The graphical notation of Petri nets allows an easy and intuitive construction of models of biological systems. To characterize the structure, behavioral properties, and dynamics of a model, there are several techniques to add time-dependent and space aspects as well as data and probabilistic aspects [32]. Petri nets have as their underlying structure a directed, finite, bipartite graph, typically without isolated nodes. The four main components of a general Petri net are as follows [33]:

- *places:* passive nodes that refer to conditions or local states;
- *tokens:* variable elements that represent current information on a condition or local state;
- *transitions:* active nodes that describe local state shifts, events, and activities in the system;
- *directed arcs:* connections that specify relationships between transitions and places.

Standard PN models are discrete and have no notion of time and as such are very useful for modeling processes without time or probability. To model more complex processes, extensions to the standard PN are used; in colored Petri nets (CPNs), data values are assigned features using different colors as data structure [34]; in stochastic Petri nets (SPNs) probabilities are added to the transitions [35]; other extensions such as hybrid Petri nets (HPNs) and hybrid functional Petri nets (HFPNs) allow for coexistence of both continuous and discrete processes [36].

In order to create a flexible, compact, and parameterizable model, we decided to use a CPN to model the early stages of the infection process and granuloma formation. Although standard Petri nets can be used to model parts of our problem, such as reaction processes and biochemical components, it becomes impractical to represent different levels of abstraction, when in addition, other aspects have to be taken into account such as the physical and spatial organization of the organism, from the intracellular to the intercellular level and beyond (molecular, cellular, and tissues). Colored Petri nets allow the description of several similar network structures in a concise and well-defined way, providing a flexible template mechanism for network designers. In colored Petri nets, tokens can be distinguished by their colors. This allows one to discriminate levels (molecules, metabolites, proteins, secondary substances, genes, etc.). In addition, the token colors can be used to distinguish between subpopulations of a species in different locations (cytosol, nucleus, and so on). 

For these reasons, we have chosen to model the early stages of the *Mycobacterium *infection process and granuloma formation and dissemination in terms of colored Petri nets. The process consists of phagocytosis of the mycobacteria by macrophages, migration of infected macrophages, and bacterial replication in an individual macrophage as well as the aggregation, granuloma formation, and dissemination of the infection. In the following section, we give a definition of CPN based on [34, 37] We use *B* to denote the Boolean type, containing the elements {false, true} with the standard operations from propositional and we use Type (Vars) to denote a set of types {Type (*v*) | *v* ∈ Vars} of a typed set Vars.

**Definition 1**

A *multiset m* over a nonempty set *S* *is  a  function m* : *S*→*ℕ*. An element *s* ∈ *S* is said to belong to the multiset *m* if *m*(*s*) ≠ 0, and then we write *s* ∈ *m*. The nonnegative integers {*m*(*s*) | *s* ∈ *S*} are called the coefficients of the multiset *m*, and *m*(*s*) is called the coefficient of *s*. The nonnegative integer *m*(*s*) ∈ *ℕ* is the number of appearances of the element *s* in the multiset *m*.

We may represent a multiset *m* by the formal sum:

(1) $$\begin{array}{c} \sum_{s\in S}m{\left(s\right)}^{\prime }s. \end{array}$$

By *S*_MS_ we denote the set of all multisets over *S*.

**Definition 2**

A colored Petri net is a tuple CPN = (Σ, *P*, *T*, *A*, *C*, *G*, *E*, *I*), *where *

(i) Σ is a finite nonempty set of types, called color sets;
(ii) *P* is a finite nonempty set of places;
(iii) *T* is a finite nonempty set of transitions such that
  (2) $$\begin{array}{c} P\cap T=\;\emptyset ; \end{array}$$
(iv) *A* is a finite set of arcs such that
  (3) $$\begin{array}{c} A\subseteq P\times T\;\cup T\times P; \end{array}$$
(v) *C* is a color function; it is defined from *P* to Σ;
(vi) *G* is a guard function; it is defined from *T* to expressions such that
  (4) $$\begin{array}{c} \forall t\;\in T:\left[\text{Type}\;\left(G\left(t\right)\right)=B\wedge \text{Type}\;\left(\mathrm{Var}\;\left(G\left(t\right)\right)\right)\subseteq \Sigma \right]; \end{array}$$
(vii) *E* is an arc expression function; it is defined from *A* into expressions such that
  (5) ∀a∈A:[Type (E(a))=C(p(a))MS    ∧Type (Var⁡(E(a)))⊆Σ],
  where *p*(*a*) is the place component of *a*;
(viii) *I* is an initialization function (initial marking); it is defined from *P* into closed expressions such that
  (6) $$\begin{array}{c} \forall p\in P:\left[\text{Type}\;\left(I\left(p\right)\right)={C\left(p\right)}_{\text{MS}}\right]. \end{array}$$

In general, a marking *m* is a function associating with each place *p* a multiset *m*(*p*) of colors (tokens) from *C*(*p*). Markings are the global states of the colored Petri net.

The Petri net semantics describes the behavior of the net, based on a firing rule consisting of a precondition and the effect of the occurrence (firing) of a single transition. Whether or not a transition can fire depends on the marking of its preceding-places and the arc expression on the input arcs. A transition is enabled and is allowed to fire, if all preceding-places, are sufficiently marked and if the binding of the variables that appear in the arc expressions evaluates to a multiset of token colors that is present on the corresponding input place. The guards of the transition should evaluate to true for the giving binding. If a transition has no preceding-places, it is always enabled. When a transition occurs with a given binding, a multiset of colored tokens are taken from each preceding-place and added to later-places in accordance with the arc expression on the arc leading to those places. Repeatedly firing transitions lead to firing sequences and determine the state space of the Petri net [33].

### 2.3. Software and Hardware Platform

Several tools are available to model biological systems using Petri nets, simulate their dynamic behavior, and analyze their structure. The Snoopy software provides an extensible, adaptive, and multiplatform framework to design, animate, and simulate Petri nets [38]. Its design facilitates the modular implementation of our CPN model allowing future extensions to be added through hierarchical organization of Petri nets. We have used the Snoopy software to implement and animate our net with two different operating systems (OS): Windows 7 (HP Intel core i7, 4 Gb RAM) and Mac OS 10.6 (MacBook Pro Intel core i7, 4 Gb RAM). The main difference between the two platforms is the additional features in the user interface for the Windows implementation. The CPN model runs with the same accuracy on both OS versions. This illustrates the platform independency of the Snoopy software framework.

## 3. Results

We have modeled the role of the innate immune system in the early stages of a *mycobacterial* infection. Our approach is to provide a large-scale model that drives the infection behavior. We have used the Snoopy tool, a framework for modeling and animating/simulating hierarchical graph-based formalisms [38], in order to create a qualitative colored Petri net representing the relevant phases in the infection process as depicted on Figure 2. In the following sections, we present the color sets Σ, places *P*, transitions *T*, and the initial marking *I* present in our CPN = (Σ, *P*, *T*, *A*, *C*, *G*, *E*, *I*).

### 3.1. Set of Color Sets Σ

We have defined five simple color sets: position, individual, status, and count and four compound color sets: macrophage, bacteria, proliferation, Granuloma composed of the basic color sets. They represent empirical information from the infection process:

- position is an integer value representing the location of a macrophage, bacteria, and/or granuloma;
- individual is a string value (mm, mac) used to identify bacteria and macrophages;
- status is a Boolean value; it can represent the infection status (healthy/infected) of a macrophage or the saturation of a proliferation;
- count is an integer value representing a threshold for the simulation;
- macrophage is composed of position, individual, and status colors and represents host macrophage immune cells;
- bacteria is composed of position and individual colors and represents *M*. *marinum* bacteria that will be injected;
- proliferation is composed of count, individual, and status colors and represents the amount of infected aggregated macrophages;
- granuloma is composed of position, individual, and count colors and represents granulomas with the amount of macrophages.

### 3.2. Set of Places *P*

The set of places of our CPN is defined as

(7) P={Infection,ImmuneSystem,Phagocytosis, Migration,BactGrowth,Checkpoint, Condition,DeadMacrophage, RecruitmentCount,AgregationAmount, StopSignaling,Maturation,Dissemination}.

They represent population of cells and multicellular complexes that are part of our model:

(i) *C*(Infection) = {Bacteria}*:* a place with the mycobacteria that intrude the host;

(ii) *C*(ImmuneSystem) = {Macrophage}: a place containing the immune cells (healthy macrophages) that will react to an infection signaling;

(iii) *C*(Phagocytosis) = {Macrophage}*:* a place containing the infected macrophages;

(iv) *C*(Migration) = {Macrophage} and *C*(BactGrowth) = {Proliferation}: places containing information about the bacterial replication within one macrophage and its movement;

(v) *C*(DeadMacrophage) = {Macrophage} and *C*(AgregationAmount) = {Granuloma}*:* places containing dead macrophages and the aggregation of recruited healthy macrophages (granuloma);

(vi) *C*(Maturation) = {Macrophage} and *C*(Dissemination) = {count}*:* places containing information about the infected aggregated macrophages (intracellular bacterial spread) and the control of the infection dissemination;

(vii) *C*(Checkpoint) = {status}, *C*(Condition) = {status}, *C*(RecruitmentCount) = {count} and *C*(StopSignaling) = {count}*:* places controlling the flow of the simulation.

### 3.3. Set of Transitions *T*

The set of transitions of our model is defined as

(8) T={BacSignaling,MacSignaling,IntracelullarSpread,Spread,t1,t2,t3,t4}.

They describe important events that govern the infection process and refer to the molecular interaction, signaling reaction and intracellular changes; they also regulate some thresholds that control the simulation:

- BacSignaling represents the signaling process when bacteria reach the host;
- MacSignaling represents the signaling process of an infected macrophage after its death (recruitment of healthy macrophages);
- IntracelullarSpread represents the bacterial replication among the aggregated macrophage in the granuloma;
- Spread represents the dissemination of granuloma infection;
- *t1, t2, t3*, *and t4* represent the control thresholds of the simulation.

### 3.4. Initial Marking *I*

The initial marking in our model determines for each place the number and type of colored tokens initially present in the places. We have the condition markings that are fixed and used to control the process and the example markings which are used in our example and can be modified without changing the workflow. They are defined as follows.

Condition markings:

- *I*(Checkpoint) = 1′(true): initialized for checking if the bacterial replication inside the macrophage reaches its limits;
- *I*(RecruitmentCount) = 1′(0): initialized for counting the number of macrophages recruited to aggregate into the dead macrophage;
- *I*(BactGrowth) = 1′(1, mm, true): initialized to trigger replicating the bacteria inside the macrophage;
- *I*(Dissemination) = 1′(0): initialized to keep count of the dissemination of the granuloma;
- *I*(Condition) = 1′(true): initialized to enable one infected macrophage become dead and start the signaling process.

Example markings:

- *I*(Infection) = 1′(1, mm) + 1′(2, mm) + 1′(3, mm) defines the initial concentration of the mycobacteria that will intrude the host. We have defined three different positions to represent different injection sites;
- *I*(ImmuneSystem) = 1′(1, mac, false) + 1′(2, mac, false) + 1′(3, mac, false)+⋯+1′(10, mac, false) defines the initial concentration of healthy macrophages in the host. The positions and amount of healthy macrophages are empirical and used just to represent their presence in the host.

All other places are initially empty, that is, there are no tokens at the onset.

### 3.5. Implementation and Execution of the Model

Our model is motivated by the biology discussed in Section 2, and it specifically focuses on the process of granuloma formation and infection dissemination. The environment of the model represents the innate immune response based on the *Mycobacterium marinum *infection process in the zebrafish embryo, although at this level, the CPN model can be used to describe the early immune response to any kind of mycobacterial infection process. The elements of the Colored Petri Net described in the previous sections represent key factors involved in the processes of infection, innate immune response, and granuloma formation. The rules of the model represent the biological interactions as described in Section 2.1, that is,

- signaling of intruding bacteria detected by healthy macrophages followed by phagocytosis;
- migration and intracellular bacterial replication within infected macrophages and their death;
- recruitment and migration of healthy macrophages in response to the dead macrophage signals;
- the aggregation process and granuloma formation;
- the bacterial spread in the aggregate macrophage and the infection dissemination.

**Figure 3:**
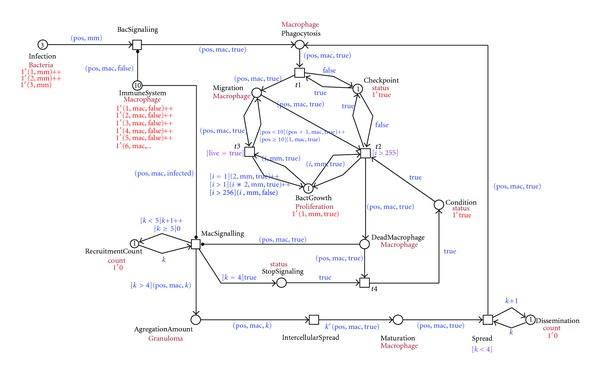
Screenshot of the CPN modeling the early stages of the immune response to the mycobacterial infection process implemented in Snoopy software.

Figure 3 shows the prototype model in a colored Petri net implemented using the Snoopy software [38]. Arrows labeled with a black dot as an arrow head are so-called testing arcs: they represent two arcs in opposite directions between the place and transition with an identical arc expression; however, the tokens are not consumed, just tested for their presence. Next, we will discuss the colored Petri net model in more detail.

As initial conditions to our model, we have defined some numbers as boundaries to check the behavior of the net using the simulation mode in the Snoopy software. The intracellular bacterial spread is limited to a concentration of 255 bacteria. In the literature, no specific information was found about the capacity of a macrophage or about its absolute position. In early stages of the zebrafish embryos, it is known where the macrophages are not present [6]. For this reason we have defined 10 relative positions to represent the presence of macrophages and their movement during the infection process and granuloma formation. In order to keep the model straightforward, we also limit the concentration of aggregated macrophages (cf.  Figure 5). Next, we have defined a threshold concerning the infection dissemination; that is, we limit the concentration of dissident macrophages that are released from the granuloma. Although from *in vivo/in vitro *experiments it seems that the dissemination is regulated by the adaptive immune system [5, 15], we have not considered this to be in the scope of our model.

**Figure 4:**
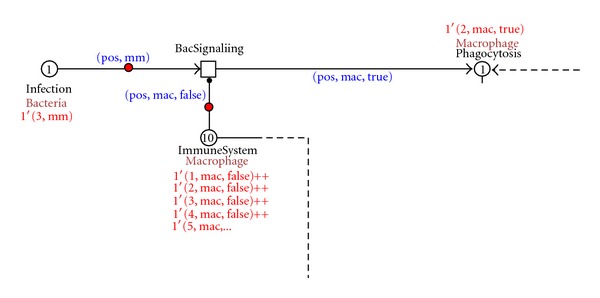
Screenshot of the infection detection and phagocytosis process.

The infection starts when the mycobacteria intrude the host. In our model we concentrate on three different positions of the mycobacteria (1, mm), (2, mm), (3, mm). Each position represents different injection sites used in the experiments with the zebrafish animal model (yolk, caudal vein, or hindbrain ventricle). In our example, the bacteria are detected by the innate immune system by signals to immune cells, in our model healthy macrophage (1, mac, false), (2, mac, false), (3, mac, false)…(10, mac, false), to take up the bacteria (phagocytosis).  Figure 4 shows this process.

**Figure 5:**
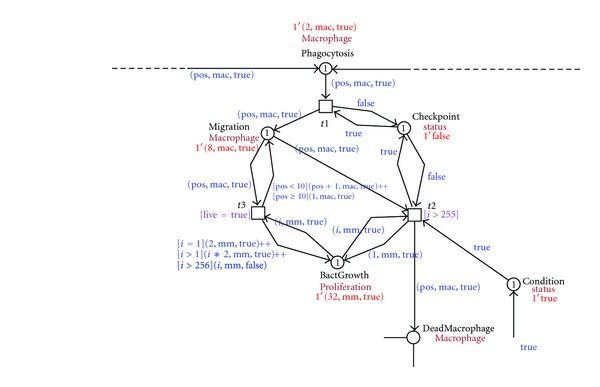
Screenshot of the migration and bacterial replication within macrophage causing its death.

After phagocytosis, the bacteria start to proliferate and move within the macrophage; the macrophage changes its position, moving to deep tissue while the bacteria replicate inside the macrophage. The intracellular growth of mycobacteria is modeled as bacterial multiplication until a concentration of 255, causing the death of the macrophage.  Figure 5 depicts this process.

**Figure 6:**
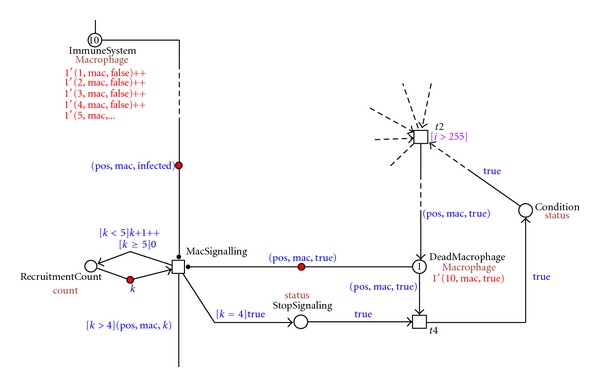
Screenshot of the dead macrophage signaling and aggregation process.

A dead macrophage starts to signal, recruiting new healthy macrophages to take up the infected macrophage and the bacteria. In this way aggregates of immune cells are formed. The aggregates contain the bacteria but are unable to get rid of them. This process is visualized in Figure 6 where a dead macrophage 1′(10, mac, true) is recruiting new macrophages to aggregate. The recruitment of macrophages is controlled by the MacSignaling transition that stops when four healthy macrophages are recruited. The numbers of macrophages that are recruited are set such that a minimal number will give rise to the formation of a granuloma. The latter is important in the development of the infection and the disease in general. The number can be increased if a particular scenario for an *in silico *experiment so requires. It will not alter the general layout of the net rather creating different balances. The place RecruitmentCount controls that.

**Figure 7:**
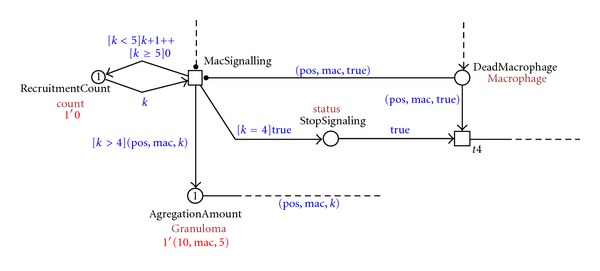
Screenshot of the granuloma formation process.

As these aggregates grow, structures develop that are referred to as tuberculous granulomas, lumps of immune cells that surround the infection.  Figure 7 shows the representation of this process in our model, where one granuloma is formed at the position 10 with a concentration of five macrophages 10 1′(10, mac, 5).

**Figure 8:**
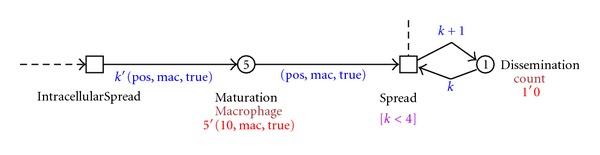
Screenshot of the intracellular mycobacterial spread and the infection dissemination process.

The intracellular mycobacterial spread in the granuloma is visualized in our model by the process depicted in Figure 8. There, all five immune cells that form the granuloma on the position 10 {5′(10, mac, true)} get infected and start the process of dissemination.

**Figure 9:**
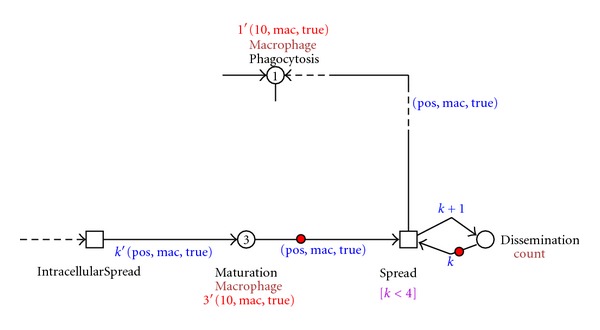
Screenshot of the granuloma formation process on the dissident infected macrophages on different positions.

In the dissemination process, an infected macrophage leaves the granuloma structure {3′(10, mac, true)} and starts another infection, moving, hosting an intracellular mycobacterial replication, dying, and repeating the granuloma formation process on another position. This process is visualized in Figure 9.

The outcome of our model reproduces the early stages of the mycobacterial process and the innate immune response. We used the animation mode available in the Snoopy software to verify the dynamic behavior of our model. This property allows to animate the token flow of the net as well as to observe the causality of the model and its behavior. For inspection and perusal, the animation sequence can be found at http://bio-imaging.liacs.nl/galleries/cpn-mmarinum.

## 4. Conclusion and Discussion

A systems' biology approach, integrating both modeling and experimental aspects, has much to contribute to the study of host-pathogen interactions. Biological processes that are relevant to the immune response occur at different scales or levels of resolution, that is, molecular, cellular, and tissue levels [39, 40]. Development of multiscale, multicompartment models based on *in vivo/in vitro *experimental data is essential to create a computational system that reflects this biological behavior [40]. In our previous work [31], we provide a basic layout addressing the modeling challenges from the integration of imaging analysis data and the Petri net formalism in different levels of abstraction, from epidemiological to genetic levels in a multiple-scale model. 

The aim of this work is to introduce a modeling approach new to the modeling of the innate immune response in a model; this modeling represents the dynamic behavior of the mycobacterial infection process. We consider our model to represent a high level of abstraction in which the infection process can be visualized in a large-scale model. Complex processes involving cell-cell or cell-bacteria communication can be modeled as small-scale processes and incorporated in our model. We use the Petri net formalism as a formal modeling method because of its extensible, modular, easy, and intuitive construction properties different from other and more broadly used modeling frameworks [32]. We have developed a high-level abstraction of the infection process by designing a PN by acknowledging the major processes of the *Mycobacterium* infection together with the basic actors that are involved in these processes. 

As a result, we have delivered a CPN model that expresses, at a high level of abstraction, the details that are involved in the early disease of mycobacterial infection. Information about the early mycobacterial infection process, the innate immune response, and the infection dissemination can be observed in our model. Through a parameterizable net that assembles information about the host-pathogenesis interaction phases, we can visualize the dynamics of the infection process. The scalability of our model allows extension on different levels of abstraction providing the aggregation of independent and related model hierarchically, that is, gene expression pathways, molecular process, cell-to-cell interaction events, and so forth. In this manner allowing experiments that simultaneously track molecular, cellular, tissue, organism, and population scale events, biologists have greatly appreciated the visualization of the processes through the animation of the PN.

Several reliable tools have been developed to create and investigate qualitative and quantitative properties of Petri nets by structural analysis, simulation of time-dependent dynamic behavior, and model checking. In the research presented here, we have chosen the Snoopy software [38] to implement and animate our model. This software is extensible and adaptive through support of simultaneous use of several models. Moreover, it is platform independent. Further extensions are to investigate the quantitative properties of the process. Such can be accomplished using the Charlie tool [41] so as to verify and validate the net and further analyze our model.

In summary, we have developed a straightforward model to explore the early mycobacterial infection and the immune response. Modeling the steps that regulate the infection process requires further testing on both theoretical and experimental levels. The results of these *in silico* experiments/findings can become the input for further analysis. It will support, for example, identification of key parameters or mechanisms, interpretation of data, or comparison of the capability of different mechanisms to (re)generate the observed data. Finally, a model that successfully describes existing experimental data may be used in the prediction of results from new experiments and generation of further hypotheses about the immune system response to mycobacterial infection helping to unravel the mechanisms of TB infection [42]. In this manner it can contribute to treatment. As indicated from the design of our CPN, the next steps in the development of the net are to add lower-level processes representing the tissue, cellular, and molecular interactions relevant to the infection process. The CPN accommodates this as hierarchical layers. Along with these layers, numerical data will become available that will allow to elaborate on the quantitative aspects of this process. The interplay of hierarchical levels and quantitative information has the potential to develop to a powerful tool for the research in tuberculosis disease, and hopefully it will further mature in a paradigm for integrated research to infection diseases.
